# Specific Trends in Pediatric Ethical Decision-Making: An 18-Year Review of Ethics Consultation Cases in a Pediatric Hospital

**DOI:** 10.1007/s10730-024-09524-7

**Published:** 2024-02-28

**Authors:** Yaa Bosompim, Julie Aultman, John Pope

**Affiliations:** 1 Northeast Ohio Medical University, Rootstown, OH USA; 2 Akron Children’s Hospital, Akron, OH USA

**Keywords:** Ethics consultation, PICU, Pediatrics, Decision-making capacity, Autonomy, Ethics committee, Rights

## Abstract

This is a qualitative examination of ethics consultation requests, outcomes, and ethics committee recommendations at a tertiary/quaternary pediatric hospital in the U.S. The purpose of this review of consults over an 18-year period is to identify specific trends in the types of ethical dilemmas presented in our pediatric setting, the impact of consultation and committee development on the number and type of consults provided, and any clinical features and/or challenges that emerged and contributed to the nature of ethical situations and dilemmas. Furthermore, in reviewing clinical ethics consultation trends for nearly two decades, we can identify topic areas for further ethics education and training for ethics consultants, ethics committee members, and pediatric healthcare teams and professionals based on our experiences. Our study with nearly two decades of data prior to the COVID-19 pandemic can serve as groundwork for future comparisons of consultation requests and ethics support for pediatric hospitals prior to, during, and following a pandemic.

The primary goal of pediatric ethics consultation is to enhance the quality and delivery of healthcare through the identification, examination, and resolution of ethical issues, questions, or concerns. Our consultation at our tertiary/quaternary hospital has increasingly become a vital asset since 2001 with the advancement of medical technologies and interventions and more complex ethical problems and issues to be understood and resolved. We have had few consults spanning the 18 years prior to the COVID-19 pandemic but are equally seeing important trends over time that help inform best practices in clinical consultation, and pediatric care delivery.

Despite the known value ethics consultation services bring to pediatric clinical practice (Larcher et al., 1997), literature describing the nature of consults is sparse with variable numbers of consults reported. In the United States, about 90% of hospitals report less than 25 consults yearly (Johnson et al., 2015; Fox et al., n.d.) with some, such as our pediatric hospital, reporting 1–10 consults annually (Johnson et al., 2015; Kesselheim et al., 2010). Following a 2020 survey, *The Children’s Hospital Association Annual Benchmark Report*, Meaghann S. Weaver and colleagues (2023) concluded that one-third of children’s hospitals lack pediatric consultation services and that future research is needed to understand these barriers.

In their review article, Carter, and colleagues (2018) speculate that “most discussion of ethical issues takes place outside of formal ethics consultation” in what they describe as “moral spaces,” a term coined by philosopher, Margaret Urban Walker (Carter et al., 2018, p. 99). In other words, most of the consults are informal or resolved among healthcare professionals holding individual or small team-based conversations. Carter and colleagues believe the few consults that make their way to the ethics committee as formal consults are the more complicated situations that cannot be resolved in these types of moral spaces and have several conflicting values and disagreements that require the expertise of clinical consultants and ethics committee members (Carter et al. 2018). In recognizing and valuing the importance of these moral spaces for ethical deliberation and resolution, it is critical healthcare providers are equipped with the appropriate tools and resources to navigate ethical dilemmas and identify the best possible action(s) or decision(s). It is equally important for healthcare teams and organizations to support the efforts of expert ethics consultants and well-trained committee members who can address complex ethical challenges in clinical care and provide recommendations resulting in better health outcomes and patient care.

Recognizing that not all ethical challenges or dilemmas are approached or resolved similarly among knowledgeable and well-trained ethics consultants, future research comparing what occurs within and external to moral spaces (bedside discussions and informal and formal ethics consults) is needed for identifying the nature and trends of challenging ethical situations and dilemmas and how best to address, if not prevent, them. In describing their processes of consults, Ramsauer and Frewer (2009) offered important recommendations for pediatric hospital ethicists and ethics committees potentially impacting how ethical dilemmas could be approached similarly across clinical sites. However, as Johnson et al. (2015) elicit, Ramsauer and Frewer (2009) do not provide an organized review of consultation content. Such a review of the content is beneficial for identifying different consultation approaches or processes, especially given the varying needs and relationships among patients, families, and providers, as well as ways to improve ethics training and education among ethics consultants and other health professionals. However, little research has been done to understand the trends of pediatric consults let alone what occurs in moral spaces, and “knowing the number of ethics consults tells us little about what impact case consultation has had on clinical care and how it was perceived by all those involved… outcomes that would seem far more important than the bare number of referrals” (Kilham et al., 2015, p. 2). Furthermore, Kilham et al. (2015) report, “it is vital that clinical ethics consultation services measure more comprehensively the range of outcomes that may follow consultation, including the response to consultation and the opinion of the child, if old enough to express it, and of the family” (p. 2).

Thus, this study aims to add to the sparse literature and promote future studies for understanding trends in pediatric ethical dilemmas that prompt informal and formal ethics consults and require expert ethics deliberation. To first understand the nature and trends of pediatric ethical dilemmas as addressed through informal and formal consultation work, we examine nearly two decades of consultation case reports, recommendations made by ethics consultants and the ethics committee, and reported outcomes.

Prior to 2001, our pediatric hospital had an informal ethics committee without a consultation service. The consultation service developed in 2001 addressing and documenting ethical challenges in pediatric care. Emerging as a 24-person committee with an on-call consultation service (typically 4–5 members from the ethics committee), our ethics team today contributes to hospital community ethics education, policy development and implementation, and support to patients, families, and healthcare teams through informal and formal ethics consultation. Despite the extensive ethics work our committee members provide, they are full time healthcare providers, academic professionals, and community members who are volunteering their time; we do not have a dedicated full-time ethicist at our institution.

Although we have relatively few consults each year (an average of 6–8 formal consults a year since 2015), many of these consults were complex, requiring multiple interviews and meetings, and ultimately enhanced consultants’ and medical team members’ ethical knowledge and skills. Many of our documented ethics cases contributed to the resolution of disputes among healthcare teams, families, and others, and offered ethical justifications based on the careful examination of values, interests, and goals of all involved in a particular situation or dilemma.

As we have emerged as a consultation service that values shared decision making, it was critical to understand the nature of our consults, and how we can continue to evolve as a consultation committee. Thus, the three primary goals of our study are to identify trends and themes of the 74 consults of our hospital; to understand the nature of our consults and how to establish best practices and education based on emerging trends, and why those trends emerge.

## Methods

### Background

Our study takes place in a 365-bed tertiary/quaternary pediatric hospital in Akron, Ohio. Prior to 2001, there was not a formal mechanism in place for consultation. The Ethics Committee (EC) of our hospital, Akron Children’s Hospital, has been addressing issues of ethics, ethical care of patients, and providing ethical leadership since 1985. Because the processes of providing these services have changed over time, from 2001 to 2019 there has been a steady increase in the number and diversity of consults in our hospital. There has been a total of 74 consults. Eleven of these consults are informal/inquiries. During the early years, most of the ethics consults were conducted by 1–2 individuals until 2014 when the ethics committee grew (27 professionals from various medical and academic specialties) and an ethics sub-committee of 4 to 6 members conducted the consults. Additionally, since 2014 ethics committee and hospital-wide presentations, Grand Rounds ethics discussions, and committee members’ attendance at local and national conferences (e.g., Bioethics Network of Ohio, American Society of Bioethics and Humanities) expanded ethics education and training for hospital staff in the effort to identify ethical issues and guide ethical decision-making.

Johnson et al. (2015) indicated that despite a vast literature on ethics consults and ethical issues that predominate in adult care settings, little is known about the ethical issues in pediatric settings and the concerns that prompt consultation requests. Thus, to better understand the utilization of our ethics consultation service and to identify those ethical issues that prompt consultation requests, we posed the following research questions: what situations might trigger formal ethics consults? Who was more likely to request a consultation? In this study, we examined the nature of the ethical questions being asked, who is asking for ethics consultation, and the ethical issues presented over time, paying particular attention to trends among clinical departments and issues presented, as well as the processes by which consults have been conducted for nearly 20 years.

### Study Design

This was a retrospective chart review of 74 ethics consults at Akron Children’s Hospital. All formal and informal consults completed from January 1, 2001 to July 1, 2019, were included in this study. There were no exclusion criteria. Data was recorded in REDCap based on an original intake form and the currently used 3-page consultation form that was established in 2001. Both the original and updated consultation forms require patient demographics, the requestor of the consultation, the ethical question(s), and the case presentation. A REDCaP database of consultation reports was created for this project and for maintaining future ethics consultation reports at our hospital.

### Analysis

Consultation data was qualitatively analyzed using thematic analysis and quantitatively analyzed using descriptive statistics (Alholjailan, 2012; Vaismoradi et al., 2013; Nowell et al., 2017). We achieved inter-rater reliability by having two investigators conduct separate analysis and then compare results. The utilization of REDCap and the retrospective entering of consult data allowed for a standardized evaluation of consult data, and the identification of any gaps or issues and address these going forward. The primary endpoint of this study was to emphasize trends, that will help guide future clinical ethics consultation and committee work as potential future ethical conflicts and issues may be predicted based on these trends.

## Results

### General Characteristics

A total of 74 consults were recorded in REDCap based on an original intake form and analyzed using thematic and descriptive statistics. Of note, requested consults for patients were primarily (97%) inpatient (Table 1), with 46% of consults requested by PICU physicians (*n* = 34). Interestingly, no consults were recorded in 2005 and 2008, with 2016 (*n* = 9) having the highest consult occurrence (Table 1). Less than 25% of consults (*n* = 16) were called by non-physician medical teams and parents/legal guardians (Table 1).

**Table 1:** General consultation characteristics

Consultation by year	n (%)
2001	1 (1%)
2002	6 (8%)
2003	2 (3%)
2004	3 (4%)
2005	0
2006	3 (4%)
2007	3 (4%)
2008	0
2009	6 (8%)
2010	4 (5%)
2011	6 (8%)
2012	6 (8%)
2013	3 (4%)
2014	2 (3%)
2015	1 (1%)
2016	9 (12%)
2017	7 (9%)
2018	6 (8%)
2019	6 (8%)

Consultation types	n (%)
Formal	63 (85%)
Informal	11 (15%)

Requestor role	n (%)
Physician (attending/resident)	58 (78%)
Parents/guardians	6 (8%)
Nurse practitioner	4 (5%)
Social worker	3 (4%)
Nurse	3 (4%)

Patient location at the time of initial consultation	n (%)
PICU	34 (46%)
Adolescent medicine	7 (9%)
NICU	6 (8%)
Neurology/neurosurgery	6 (8%)
Psychiatry	3 (4%)
Hematology/oncology	3 (4%)
Burn unit	3 (4%)
Fetal treatment center	2 (3%)
Outpatient	2 (3%)
Other (med/surg, genetics, cardiology)	1 (1%)

*No consultations were reported in 2005 and 2008

### Pediatric Patient Characteristics

Approximately 27% of consults (*n* = 20) involved patients with neurological pathology (brain and spinal disorders/injuries). Behavioral health disorders comprised 16% of the cases (*n* = 12) (Table 2). Only 15% of consults involved patients with blood diseases and cancers/tumors despite these common issues within ethics consults in adult populations (Table 2).

**Table 2:** Pediatric Patient Characteristics

Age	n (%)
< 1 year	16 (22%)
1–4 years	11 (15%)
5–11 years	14 (19%)
12–15 years	13 (18%)
16–18 years	8 (11%)
18+	9 (12%)
not reported	3 (4%)

Gender	n (%)
Male	37 (50%)
Female	32 (43%)
not reported	5 (7%)

Pathology observed	n (%)
Neurology	20 (27%)
Behavioral health	12 (16%)
Pulmonary	10 (14%)
Trauma (burns/injuries/abuse)	9 (12%)
Developmental/genetic	8 (11%)
Malignancy	8 (11%)
Cardiovascular	7 (9%)
Hematologic	7 (9%)
Others (infections, kidney disease)	3 (4%)
Gastrointestinal	3 (4%)
Rheumatologic	1 (%)

### Ethical Considerations and Outcomes

The ethical considerations of the consults were the result of qualitative analysis of the ethical question(s) posed, the case presentation, and recommendations. Over 60% of consults (*n* = 43) involved ethical questions about decision-making capacity. Of note, over 50% of consults (*n* = 38) involved questions about refusing/forgoing life sustaining treatments (Table 3).

**Table 3:** Ethical Considerations and Outcomes Following Consultation

General ethical issues under consideration in consults	n (%)
Decision making capacity	43 (61%)
Refusal/foregoing life-sustaining treatment	38 (54%)
Pediatric assent/parental decision	36 (51%)
Pain and Suffering	32 (45%)
Quality of life/palliative care	30 (42%)
Quality of care/treatment	22 (31%)
Medical futility/non-beneficial treatment	21 (30%)
Discharge planning	11 (15%)
Medical neglect	7 (10%)
Informed consent	6 (8%)
Resource allocation/utilization	6 (8%)
Brain Death	6 (8%)
Palliative care	4 (6%)
Miscommunication/misunderstanding	3 (4%)
Truth telling	1 (1%)

Ethical themes	n (%)
Rights of the parents	37 (52%)
Rights of the hospital/provider	31 (44%)
Right of the patient	19 (27%)
Delaying/interfering with treatment	8 (11%)
Comfort	7 (10%)
Conflicting wishes	7 (10%)
Child abuse	6 (8%)
Natural death	6 (8%)
Alternative/complimentary treatment	6 (8%)
Safety of patient	6 (8%)
Medical distrust	5 (7%)
Uncertain prognosis	5 (7%)
Religion/cultural conflict	5 (7%)
Communication barriers	4 (6%)
Preserving life	4 (6%)
Organ donation	4 (6%)
Seeking second opinion	3 (4%)

Actions taken by ethics committee	n (%)
Provide recommendation/advice	68 (92%)
Conflict resolution/mediation	36 (49%)
Improved understanding of patient values	26 (35%)
Provided information/referrals (palliative, Children services)	11 (15%)
Emotional support for family	11 (15%)
Emotion support for patient	6 (8%)
Connection to resources	4 (5%)
Validation/supporting health team recommendations	3 (4%)
Legal intervention	2 (3%)

Interpersonal conflict type	n
Treating team vs. family/guardian	39
Treating team vs. patient	9
Treating team vs. specialist	1
Parent/guardian vs. patient	1

Over 50% of the emerging ethical themes focused on the rights of parents (*n* = 37) and over 40% focused on the rights of the hospital and the healthcare providers (Table 3) in continuing or discontinuing care (*n* = 31). Actions taken by the ethics committee primarily involved providing recommendations/advice (92%) and conflict resolution (49%) respectively. Most of the interpersonal conflicts presented in the consults involved treating teams vs. the family (*n* = 35).

## Discussion

In conducting a thematic, quantitative analysis of consults at our hospital to examine the nature and structure of ethics consult services pertinent to neonatal and pediatric populations, we focused on the ethical questions being asked, who is requesting an ethics consultation, and the ethical and clinical issues that emerged during the consult. Additionally, we examined how consultation processes changed over a span of nearly 20 years and what gaps still need to be filled.

### Why So Few Ethics Consults?

For the number of consults per year, our data showed inconsistencies yearly, with an average of 4 consults per year. No consults were reported for 2005 and 2008. Our data is consistent with the current literature. Evidence shows that in the United States, the number and frequency of ethics consults in pediatric hospitals differ, with about 90% of hospitals reporting less than 25 consults yearly (Johnson et al., 2015; Fox et al., n.d.), and with some even reporting 1–10 consults annually (Johnson et al., 2015; Kesselheim et al., 2010). So why did our hospital receive so few consults, considering the possible occurrence of countless ethically and clinically challenging cases over the 18 years?

Families and healthcare providers are constantly making decisions about “whether to continue life-prolonging treatment or to shift to palliative care” (Carter et al. 2018; Janvier et al. 2017; Racine & Shevell 2009). As supported by our results, the two most common ethical issues under consideration in consults were decision-making capacity and forgoing-life-sustaining treatment, respectively (Table 3), which should have stimulated more consults. The presence of interpersonal conflicts between families and treating teams (*n* = 39) about the best possible outcome for patients should trigger an ethics consult, especially since the triad of stakeholders (patient, family, provider) may reveal value conflicts even when each party has the best interest of the patient in mind, especially when it comes to decisions on whether to forgo treatment (Bluebond-Langner et al., 2007).

In the case of our hospital, there was no formal consultation service before 2001 and most ethics consults at Akron Children’s were held informally early on. However, there was an increase in the frequency of ethics consults with the establishment of the formal ethics committee and the standardized 3-page consultation form.

We suspect we see few consults because multi-disciplinary healthcare professionals, patients, and parents and guardians are unaware of or know how to utilize our ethics consult service, i.e., contact hospital ethicists or ethics committee members. This is consistent with our data because we saw that almost 50% of consults came from the PICU (Table 1) and over 50% of the consults were requested by physicians (Table 1). Interestingly, our data is similar to those trends observed in recent pediatric consultation studies (Carter et al. 2018; Leland et al., 2020; Streuli et al. 2014; Winter et al. 2019).

In studying the difficulty of parents and guardians contacting ethicists at 190 children’s hospitals, Sharma et al. (2022) found that it took three separate contact attempts to find an ethicist or the right person who could assist; waiting time could be several minutes to several days. At Children’s Hospital of Philadelphia, with a high-volume pediatric ethics consultation service, only 5.7% of 245 consults in a period of 5 years (2013–2018) came from parents (Nathanson et al., 2021). Better visibility in social media, on web pages, and hospital directories is important as well as educating patients and families of ethical services to assist in navigating through challenging ethical dilemmas.

We also consider that healthcare professionals may not be identifying ethical dilemmas in their practice, despite the presence of such dilemmas, or they might think calling a consult is inconvenient (Navin et al., 2020). Alternatively, healthcare professionals might be receiving better undergraduate and graduate training in ethics, professionalism, and health law, and are able to navigate ethical conflicts without resorting to an informal or formal ethics consultation (Navin et al., 2020). Even so, many times we observe that healthcare professionals get overwhelmed with clinical ethical challenges and have identified an increase in the complexity of clinical and ethical situations with growing trends in such areas as decision-making authority and capacity. Studies have shown that 90% of pediatricians who have experienced ethics consults found them to be quite helpful (Morrison et al., 2015). Even though few consults are reported in our study (*n* = 74), we can examine these trends and the ethical challenges that give patients, families, and healthcare teams pause, while identifying opportunities for further pediatric ethics consultation refinement.

### Ethics Consult Requests

Studies have examined factors contributing to low ethics committee utilization, particularly among physicians (Kesselheim et al., 2010; Thomas et al., 2015) due to factors such as having little or no access to consultation services (Morrison et al., 2015). However, our findings showed most consult requests came from physicians. In discovering less than 25% of consults were requested by nonphysicians, parents/legal guardians, and medical teams (Table 1), this data is consistent with previous studies discussing ethics consult service characteristics in children’s hospitals (Henriksen Hellyer et al., 2015).

A study by Zhao et al. 2022a investigating the perceptions and barriers existing in the utilization of ethics consulting services within a treatment team revealed that physicians are more likely to use ethics consult services because they are the best educated on the process of an ethics consult (Zhao et al. 2022a, 2022b). They also concluded that since nonphysician members of the treatment teams have direct interaction with patients (and loved ones) regularly, they have a unique understanding and perspective of the ethical dilemmas and must be encouraged to call consults when appropriate. However, as Zhao et al., 2022b note, the power dynamic between attending physicians and other team members can diminish team members’ voices and give them the impression that the team lead must sign off on patient-care decisions, including consult requests. While consultation requests should come from any stakeholder in the clinical setting, we support the view that the attending should have knowledge of the consult and of the person requesting the consult (Orr & Shelton, 2009), unless there is a compelling reason to maintain anonymity for the safety of the requestor. In such cases, we would guide the individual toward open communication, conflict resolution, and supportive resources. In the case of our pediatric hospital, equipping nonphysician team members with resources and support through ethics education and improved awareness of consultation services will institutionalize this process (Zhao et al. 2022a). We can achieve this through our ongoing efforts to deliver presentations and workshops that address our roles and responsibilities as ethics committee members and consultants, as well as being more deliberate in our web and social media presence. Additionally, encouraging physicians to be supportive of their team members in seeking out ethics guidance and requesting consults without negative repercussions can be an effective step in improving team-based, patient/family-centered ethical decision-making and minimizing counter-productive power differentials.

While many of our ethics consults guided decisions regarding who ought to have decision-making authority (e.g., parents/guardians) to best promote patients’ health and wellbeing and avoid unnecessary harm, more recent consults emphasized the importance of shared decision-making, and offered supportive care to providers, healthcare teams, and families. Despite only 6% of consults indicating “communication barriers” (Table 3), the value of clear communication and transparency in pediatric patient care was a continuous theme within ethics consultation recommendations.

### An Increase of Behavioral and Neurological Disorders

Since 2009, it is interesting to note that ethics consults involving behavioral/psychological and neurological disorders have increased and make up 43% of pathologies reported in our ethics consults with associated challenges to patient quality of life, parental/guardian and patient decision-making, and determinations of best-interests standards and quality of care. However, most of the ethical questions and themes involving behavioral/psychological disorders centered around the “rights of the hospital” (i.e., discharging patients) and whether the healthcare team has the right to continue or discontinue care (*n* = 7). Trends at the turn of the century focused on end-of-life decision-making and whether to withhold or withdraw treatments. And, while end-of-life situations do occur and are discussed in our consults, we are encountering more clinically complex behavioral and neurological situations that lead to value-based conflicts centering on treatment decisions which prompt ethics consult requests mostly by healthcare professionals caring for patients in the PICU (46% of cases).

### Decision-Making and Ethical Rights in Pediatric Ethics Consults

Our data reveals that interpersonal differences between healthcare professionals, parents/guardians, patients, personal values, and socio-economic factors all contribute to ethical dilemmas in consults and can contribute to conflicts requiring assistance from ethicists (Buchanan et al., 2019). Yes, young children lack decision-making capacity, but “the cognitive capacities needed to foster decision-making capacity develop as children age” (Buchanan et al., 2019). While we encourage and support the contributions of parents/guardians in decision-making, “the current literature supports the involvement of children in healthcare decision-making in an age-appropriate manner” (Buchanan et al., 2019).

Questions about parental rights, hospital rights, and patients’ rights, particularly in cases that stress decision-making capacity, were prominent in the consults, consistent with what we experience in pediatric ethics (Santoro and Bennett 2018). It’s necessary to note that nearly half of the primary ethical issues in the consults centered around decision-making capacity and foregoing life-sustaining treatment/nonbeneficial treatment. This trend was also consistent with the available studies analyzed. Furthermore, the data we analyzed revealed decision-making capacity as a prominent trend in the more recent consults, beginning in 2016 (Table 3).

A noteworthy case that reflected the complexity of parental rights, patient’s rights, and hospital rights in the setting of decision-making capacity involved a 12-year-old patient with significant long-term health conditions who required a life-saving procedure. An ethics consult was requested by the PICU attending because the treatment team expressed that they would be more comfortable and accepting of decisions made regarding the patient’s care if the patient was part of the discussion and decision-making. The ethical questions examined included: (1) Should a 12-year-old with significant long-term health conditions have the right to consent to care/procedures? (2) Should the mother be the decision-maker for major care options/decisions in a family with Children’s Services involvement (i.e., concerns about child abuse), and poor care adherence? In the end, the ethics committee advised reassessing parental understanding of the risk and benefits of the procedure and Children Services input about parental decision-making moving forward. They also recommended obtaining developmental consent and support for the patient.

From this case, we identified that the parents are responsible for the care of their children with authority to make clinical decisions regarding their children. However, we acknowledged the limits to this authority if the child’s best interest is not considered, or if the parent or guardian lacks decision-making capacity, and there is unnecessary risk. This brings to light the important role of shared decision-making in pediatric ethics consults, which has become more evident in our consultation reports since 2015. Ward (2013) and Schneiderman et al. (2006) noted that normalizing shared decision-making in pediatric ethics consults will not always guarantee success but can provide valuable details on aspects that lead to a failed consult (Schneiderman et al., 2006; Ward, 2013).

Similarly, pediatric providers have an ethical duty to provide standard care that meets the patient’s needs and not necessarily based on parents’ or guardians’ requests. Additionally, while research has shown that adolescent brains still lack some development in the areas that regulate cognitive control, they can understand and thoughtfully consider information and options regarding their health and potential treatments (Buchanan et al. 2019; Sanders, 2013). Streuli and colleagues encourage further research into how the creation of documentation that captures the “verbal and nonverbal expression of volition will include severely ill children in a more active and comprehensive manner” (Streuli et al. 2014) with implications of improved shared decision-making in consults.

Generally, most pediatric ethics consults involve neonates, infants, and young children who are very sick and not participating in decision-making (Streuli et al. 2014). Our study was consistent with this trend, with almost 25% of our cases involving children less than one year (Table 2). Generally, parents or guardians make decisions that reflect the child’s best interest and are consistent with the recommendations of the healthcare team.

However, a growing trend in our hospital revealed conflicts between healthcare teams and parents or guardians with reported concerns among physicians about parents’ decision-making capacity and their ability to make care-based decisions in their child’s best interest. Our findings are consistent with other studies (Orr & Perkin, 1994; Opel et al., 2009); however, decision-making capacity was a less common reason in other studies for initiating an ethics consultation (Johnson et al., 2015). Literature findings suggest reasons for initiating pediatric ethics consults vary among pediatric hospitals and a future examination of associated factors such as patient and family populations, hospital environment and culture, and provider knowledge and experience is warranted.

We found reported conflicts within ethics consultation reports indicated a lack of shared decision-making due to factors such as communication gaps, a poor understanding of cultural values, parental/guardian distrust of the medical community, and exclusion of capable adolescent patients in decision-making processes.

In their discussion of the role of children in making decisions, Buchanan et al., 2019 highlight that involving pediatric patients appropriately in decisions about their health is empowering and a way to encourage their participation in their medical care. This “contributes to their perception that they have some control and influence over their life, and results in a greater sense of self-esteem and competence, while reducing anxiety and fear of the unknown” (Buchanan et al., 2019 p. 272). Even so, some feel it is inappropriate to place autonomy over safety and engage children extensively in their medical care due to their limited cognitive development. When it comes to *parental obligation to their children*, healthcare professions must think about the “value systems of patients and families beyond prognosis alone, maintaining the essential role of the parents in shared decision making, balanced with the best interest and safety of the child” (Buchanan et al., 2019 p. 273; Orr et al., 2003; Opel, 2017), while also acknowledging the difficulty of the parental decision and reassuring good parenting (Yazdani et al. 2022).

### Caring for the Caregiver

Though not extensively detailed in consultation reports, some recommendations presented in the consults suggest offering care and support to healthcare providers. This care was often provided by individuals external to the EC (e.g., pastoral services). Treating teams typically face ethically stressful situations, so hospital ethics consultation services must continue bringing awareness and creating the appropriate measures for support. In the case example of the 12-year-old patient discussed prior, we saw that the treating team was burdened with uncertainty over who should have decision-making power. Rasoal et al. (2017), identified another important role for ethics professionals in the creation of clinical ethics support (CES) as part of the ethics consult service. CES is “the formal or informal provision of advice and support to healthcare personnel on ethical issues arising from clinical practice and patient care within the healthcare setting” (Rasoal et al. 2016, 2017; Owen, 2001; Puntillo et al., 2001; Slowther et al., 2004). Our institution can become more robust in its support of healthcare teams by considering the creation of a CES to operationalize the supportive resources the ethics team recommends or offers for treating teams dealing with morally challenging ethical consults.

### Ethics Education, Training, and Promotion of Pediatric Ethic Consults

Finally, our study has revealed that additional ethics education and training among consultation and committee members and better communication and marketing are needed to improve the quality and access to consultation services. In terms of quality improvement, how ethical dilemmas are identified and described, and ethical justifications for clearly articulated recommendations can be more detailed in future consultation reporting. We observed a significant improvement in the presentation of the ethical dilemmas, clinical case details, and recommendations with the use of a standardized form in 2006. However, depending on the consultation team and designated consult leads (typically a chair or co-chair of the ethics committee), the thoroughness of the case presentation and quality of ethical support for recommendations varied. With the growth of consults and the increasing demand for ethics consult services by healthcare providers at children’s hospitals (Leland et al., 2020) our hospital must continue to develop consistency in its ethics consultation training and practices, and post-consultation evaluation approaches to best address challenging, future dilemmas. Additionally, it is critical that consults are more accessible, available, and known within the clinical community (Morrison et al., 2015).

## Study Limitations

Consultation data was inconsistent over time due to developments in the pediatric ethics consults and consultation process. Some data from our earlier consults (2001–2006), which was contained not in a database but on hardcopy consultation reports, including illegible handwritten reports, could not be included in our analysis. Qualitative data will be varied due to such considerations as how consults were conducted, who conducted the consultation, and the type of information that was deemed appropriate at the time of the consult. Consultation data that required input from a committee improved the reliability of consultation data. The number of consults between 2001 and 2019 is relatively few for identifying significant, generalizable trends. Nevertheless, our comparative analysis of ethics literature and consultation services in comparable hospitals throughout this paper strengthens our data and contributes to ongoing discussions with variations among types of ethical issues and consultation practices.

Additionally, because this is a single-site analysis we are unable to generate generalizable data that is applicable to other institutions. Nevertheless, our descriptive and thematic examination yields further information about the types of ethical dilemmas and issues we have encountered over an 18-year period, and the challenges we face in offering our ethics consultation services to a wider population of patients, families, and non-physician providers.

## Conclusion and Future Directions

Due to the lack of requests among non-physicians, including other types of healthcare professionals and the families of patients, efforts should be made to increase awareness of the ethics consultation service and committee at our hospital through education and web and social media marketing. Additionally, non-physician team members who wish to call an ethics consult should feel empowered to do so, and comfortable enough to communicate their intentions with physician team leads. Mutual respect, humility, effective communication, and emotional support offered by physicians, especially those who might not recognize the ethical issue or understand how it impacts team members, patients, and others, can promote a positive culture of ethics consult utilization. Additionally, a deeper dive into why our healthcare teams might not call for an ethics consultation, especially when ethical issues are presented in medical notes and records, is needed. Over the span of 18 years of ethics consults less than 25% of consults were requested by non-physicians (*n* = 16). Our consultation forms should align with the fields in the new database; forms should be completed for improving our documentation (forms have been incomplete). More follow up documentation and assessment for quality improvement would also be valuable in understanding whether consultation recommendations were useful, since we no longer are implementing consultation evaluations (which were done for a very limited time about a decade ago). Additionally, encouraging the recording of the support provided to treating teams in the consults and considering the implementation of CES to offer more systematic support to the treating team, will be beneficial. Finally, the provision of dedicated time and effort from a full-time clinical ethicist could help in achieving the recommendations listed above.

## Abbreviations

CSB: Children’s Service Board; Children’s Protective Services
PICU: Pediatric Intensive care unit
NICU: Neonatal Intensive care unit
EC: Ethics committee
CES: Clinical ethics support
